# Detecting faulty lithium-ion cells in large-scale parallel battery packs using current distributions

**DOI:** 10.1038/s44172-025-00543-x

**Published:** 2026-01-21

**Authors:** Pierre Lambert, Ross Drummond, Joseph P. Ross, Eloise C. Tredenick, David A. Howey, Stephen R. Duncan

**Affiliations:** 1 Department of Engineering Science, University of Oxford, Oxford, UK; 2 École Centrale de Nantes, Nantes, France; 3 School of Electrical and Electronic Engineering, University of Sheffield, Sheffield, UK; 4 Brill Power Limited, Oxford, UK; 5 Faculty of Science and Technology, University of Canberra, Bruce, ACT Australia; 6 The Faraday Institution, Quad One, Harwell Campus, Didcot, UK

**Keywords:** Batteries, Electrical and electronic engineering

## Abstract

One of the main concerns affecting the uptake of battery packs is safety, particularly with respect to fires caused by cell faults. Mitigating possible risks from faults requires advances in battery management systems and an understanding of the dynamics of large packs. To address this, a machine learning classifier based upon a support vector machine was developed that detects cell faults within large packs using a limited number of current sensors. To train the classifier, a modelling framework for parallel-connected packs is introduced and shown to generalise to Doyle-Fuller-Newman electrochemical models. The fault classification performance was found to be satisfactory, with an accuracy of 83% using current information from only 27% of the cells. Validation on experimental pack data is also shown. These results highlight the potential to combine mathematical modelling and machine learning to improve battery management systems and deal with the complexities of large packs.

## Abstract (web summary)

The formation of faults in lithium-ion batteries can significantly impact pack safety and performance. Pierre Lambert and colleagues propose a modelling and machine learning framework to detect these faults from the current oscillations found in parallel connected packs.

## Introduction

Applications including transport electrification and grid energy storage are leading a rapid growth in the roll-out of large lithium-ion (Li-ion) battery packs^1^. As large packs become more widely adopted, concerns have been raised about their safety and the ability of the battery management systems (BMS) to detect faults that could eventually lead to fires^2,3^. Various faults can occur during operation, mainly due to ageing and improper use^4^, and several instances of electric vehicle (EV) fires caused by battery pack faults have now been reported^5^. For example, the National Transportation Safety Board reported 17 lithium-ion battery fires on Tesla vehicles in the USA in 2018, out of a total of 350,000 vehicles^6^. Detecting these faults and isolating them before they become dangerous is therefore a growing concern for EV and grid storage users and manufacturers. Each Li-ion battery pack typically has its own individual architecture (with little standardisation across the field) because each application generally has unique energy and power requirements. Cells in battery packs are typically connected in parallel within individual modules, with the modules then connected in series^7^. This arrangement is often referred to as *n*P*m*S, where *m* modules of *n* parallel cells are connected in series. Within parallel-connected modules, both external and internal faults can occur^8,9^. External faults are primarily caused by sensor and connection issues, while the causes of internal faults include overcharge, over-discharge, short circuits, accelerated degradation, and thermal runaway^10^; these form a varied collection of issues that can be challenging to detect since each cell behaves as a ‘black-box’. While the initiation of external faults can be sudden, internal faults often appear gradually, and thus their formation can be monitored and detected more effectively. The ability to detect and manage the development of internal faults (even though doing so may be challenging using existing pack sensor technology) motivates the fault-detection algorithms of this work.

Problems associated with battery pack faults, including fires and deterioration in pack performance, have motivated BMS fault-detection research. Cells in large-scale battery packs have intrinsic variability caused by slight changes in manufacturing and usage^11^. Within parallel-connected packs, this cell-to-cell variability causes an uneven distribution of currents across the branches in the pack, resulting in the appearance of fluctuations^12–14^. As the behaviour of these current fluctuations depends upon the cell-to-cell variability, current measurements can be used to understand pack health. Since current sensors are already widely deployed for state-of-charge estimation (although usually not all parallel branch currents are measured) and are relatively inexpensive, they have the potential to be scalable, cost-effective, and accessible tools for diagnosing pack health and detecting faults. However, improvements in BMS algorithms are needed to make more effective use of this data.

In response, this paper develops a fault-detection method that uses the information encoded within the uneven current distributions of parallel-connected packs. First, a model of a large battery pack is established, and the branch currents simulated. An internal cell fault-detection algorithm based upon the support vector machine (SVM) is then proposed using the simulated data. It should be noted that the use of branch current distributions as a means of detecting cell faults has recently been demonstrated by Ding et al.^15^, whose work estimated the current distributions based on terminal voltage, total pack current and state-of-charge (SoC) using a long short-term memory (LSTM) network. They compared the estimated branch currents to the actual measured signals and used a generated residual signal to detect connection faults. The main differences with our work include the fact that Ding et al. ^15^ proposed a detection method for connection faults (external faults) whereas we study accelerated degradation faults (internal faults); also, they focus on small packs (4p1s) whereas our results are based on large packs (74p1s).

The modelling framework and analysis proposed here also differs. In particular, our framework expresses the branch currents of the parallel pack explicitly in terms of the model states and the applied current. This formulation allows the differential algebraic equations (DAEs) of the pack model to be converted into a set of ordinary differential equations (ODEs), significantly simplifying the pack model equations. The obtained expressions for the branch currents are also shown to generalise to more complex battery models than the circuit models developed for the fault-detection algorithm, in particular to the Doyle-Fuller-Newman (DFN) electrochemical model based on porous-electrode theory. As far as the authors are aware, these are amongst the first simulations of parallel connected DFN models, a result that will enable detailed electrochemical simulations of Li-ion battery packs in the future.

Three main approaches have been used to detect battery faults in previous works: model-based methods, signal processing, and knowledge-based methods^16^. Model-based methods rely on battery models (mostly electrochemical or equivalent circuit) whose parameters are estimated using system identification techniques^17–19^. The measured and estimated signals are compared and a residual signal is extracted to diagnose a potential fault^20^. Signal processing methods rely on large datasets from which fault features are extracted (e.g., using entropy and wavelet transforms)^21,22^; faults are detected once a given Z-score threshold has been exceeded for a certain feature. Finally, the knowledge-based approach involves applying methods such as expert systems, fuzzy logic, and neural networks to diagnose battery faults^23–25^. Criteria describing the faulty state of the battery are established and thresholds defining the detection of a fault are determined.

The need for effective fault detection algorithms for BMS designs has motivated several studies on this topic. Tran et al.^26^ proposed a model-based sensor fault diagnosis method and demonstrated how the equivalent circuit model parameters were affected by cell degradation and by sensor faults, with the proposed method able to detect and isolate voltage and current sensor faults from the estimated cell degradation. Nuhic et al.^27^ developed a data-driven approach for health diagnosis using a SVM, which was shown to effectively learn the degradation behaviour of Li-ion cells. Yao et al.^28^ also demonstrated the ability of a SVM classifier to identify a cell fault and give an indication of its severity. Finally, Sidhu et al.^29^ proposed a model-based method to diagnose multiple faults using extended Kalman filters to represent signature-fault models.

Building on these results, the primary contributions of this article are as follows. An equivalent circuit model of a parallel-connected battery pack is developed and used to simulate single parallel modules (74p1s) within a Tesla Model S battery pack (74p96s) containing Panasonic NCR 18650B cells. An expression for the parallel-pack branch currents is derived which converts the model DAEs into ODEs. This expression for the currents is shown to hold for a broad class of cell-level models, in particular for parallel-connected DFN electrochemical battery models. The current distributions from the 74p1s pack model simulations are then analysed and their response to cell faults caused by accelerated degradation is investigated. A cell fault-detection algorithm based on a support vector machine is then trained and applied. The trained classifier was evaluated on experimental pack data, and, even though the algorithm was trained on simulation data, its ability to predict faults transferred over to experimental data. These results highlight the potential to use relatively inexpensive sensing hardware—current measurements at pack level—for fault diagnosis, improving pack safety and performance.

## Results and discussion

### Simulations

We now describe our model for parallel-connected Li-ion cells that captures the impact of variations in the series resistance *r* and capacity *Q* on the overall response of the pack. The key feature is the resolving of Kirchhoff’s laws such that the DAEs of the pack model are converted into ODEs, making it significantly simpler to solve. The class of cell-level models to which the following pack-level modelling results can be applied are those where voltage can be expressed as

1 $${V}_{k}(t)=h({x}_{k}(t))+{r}_{k}{i}_{k}(t),\quad k=1,\,2,\ldots ,n$$

where the index *k* ∈ {1, 2,  …,  *n*} relates to the parallel-connected cell number in the pack, *n* is the total number of cells in parallel, *x*_*k*_(*t*) is the dynamic state of cell *k* associated with a time derivative, *r*_*k*_ is the respective cell series resistance and *i*_*k*_(*t*) is the branch current (as in the current flowing into branch *k* of the parallel connections). Connecting these cell-level models in parallel means that Kirchhoff’s laws have to be satisfied to compute the branch currents *i*_*k*_(*t*),

2a $$h({x}_{j}(t))+{r}_{j}{i}_{j}(t)=h({x}_{k}(t))+{r}_{k}{i}_{k}(t),\quad j,k\in \{1,2,\,\ldots ,\,n\},$$

2b $${\sum}_{k=1}^{n}{i}_{k}(t)=I(t).$$

**Fig. 1:**
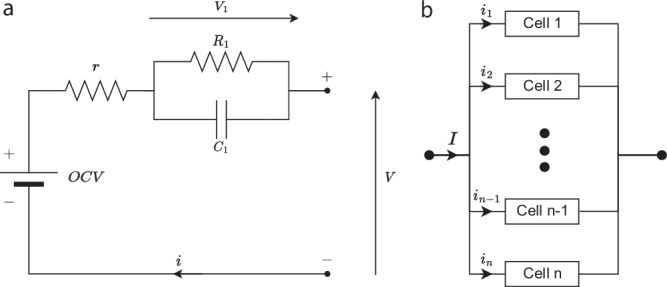
Schematic of the cell-level battery model used to build the packs. **a** Equivalent circuit model and **b** module of *n* cells connected in parallel.

Combining these with the cell dynamics means that parallel-connected pack models are described by a set of DAEs^30^. However, using the approach outlined in the Methods section, the algebraic equations of Kirchhoff’s laws from Equation 2 can be resolved to give

3a $${i}_{1}(t)={\left({r}_{1}{\sum}_{k = 1}^{n}\frac{1}{{r}_{k}}\right)}^{-1}\left({\sum}_{k=2}^{n}\frac{\Delta {h}_{k1}(t)}{{r}_{k}}+I(t)\right),$$

3b $${i}_{k}(t)=\frac{1}{{r}_{k}}\left({r}_{1}{i}_{1}(t)-\Delta {h}_{k1}(t)\right),\quad k=2,\,3,\,n,$$

where Δ*h*_*jk*_(*t*) = *h*(*x*_*j*_(*t*)) − *h*(*x*_*k*_(*t*)). With this expression, the branch currents can be written as a function of the states *x*_*k*_(*t*) (which, for equivalent circuit models such as Fig. 1, could be the state-of-charge and voltages, and, for electrochemical models, could be the potentials and concentrations) and the applied pack current *I*(*t*). Resolving Kirchhoff’s laws using Equation 3 means that the parallel-pack model does not have to numerically solve Equation 2 at each time step of the simulation to compute the branch currents *i*_*k*_(*t*). Instead, these currents are defined by Equation 3 and are expressed in terms of the states of the cell model *x*_*k*_(*t*) and the applied pack current *I*(*t*), allowing the pack model dynamics to be solved as an ODE rather than a DAE^30^. Turning the DAE parallel-pack model into an ODE also gives insight into the distribution of currents between branches in the pack, in contrast to the numerical approach where the computational solution only gives a limited insight into the mechanisms by which the pack’s currents re-distribute themselves. The numerical solution for computing the parallel-pack branch currents is, however, widely used in modelling studies^31–34^, estimator designs^35–38^ and simulations based upon the PyBAMM^39^ open-source software^40^.

The extent to which this computational solution has been used indicates the broad applicability of the analytic solution of Equation 3 to understand these nonlinear parallel-pack dynamics. Moreover, the proposed approach overcomes some of the restrictive assumptions of existing analytical solutions, such as the open circuit voltage (OCV) being affine^41,42^ and the cell-level model simply being a voltage source and a series resistor^13,43^. Instead, with Equation 3, the voltage is allowed to be of the form of Eq. (1), as in the sum of a linear series resistance term and a nonlinear function of the states. Compared to the existing derivation for the branch currents from Drummond et al.^44^, the method presented here is simpler since it does not involve computing a matrix inverse. Instead, in this work, the structure of the equations defining Kirchhoff’s laws are exploited to solve Equation 2.

The cell-level model used to design the fault-detection algorithm for the parallel-pack branch currents is the equivalent circuit model described in the Methods section. The different cell parameters were assumed to be normally distributed from cell-to-cell by extrapolating the results of Schneider et al.^45^, with the means of the parameter distributions given in Table 1 and the standard deviations given in Table 2. Since the values observed in Schneider et al.^45^ were based on different cells (Samsung INR18650-25R) from those considered here (Panasonic NCR 18650B), the *σ*/*μ* ratios from Schneider et al.^45^ were first computed and then related to the mean values from Table 1 to derive the standard deviation values for the NCR 18650B cell considered here. The standard deviations computed using this method are stated in Table 2.

**Table 1:** Mean values of the cell model parameters

Parameter	Mean Value	Unit	References
*r*	19	m*Ω*	^61^
*R*_1_	1.7	m*Ω*	^61^
*C*_1_	5598	F	^61^
*Q*	3350	mAh	^62^

**Table 2:** Standard deviations of the cell model parameters

Parameter	*σ*/*μ* ratio	Standard deviation *σ*	Unit
*r*	2.09 %	0.40	m*Ω*
*R*_1_	1.64 %	0.028	m*Ω*
*C*_1_	7.12 %	399	F
*Q*	0.28 %	9.4	mAh

**Fig. 2:**
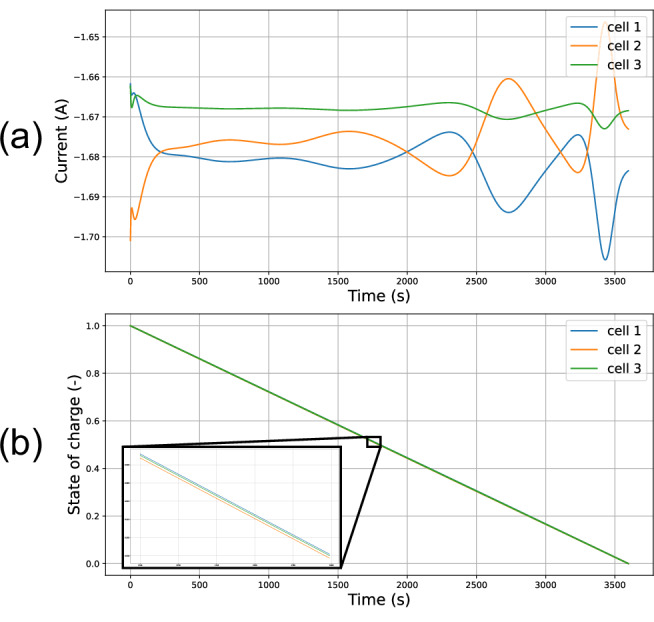
Discharging behaviour of three cells connected in parallel. Simulated currents (**a**) and states of charge (**b**) for three parallel-connected cells during a 1C discharge.

To analyse the behaviour of our model, a 1C discharge of three cells connected in parallel was considered. The simulated currents and state-of-charge values are plotted in Fig. 2, and it is noted that the current distributions for the cells are similar in shape to those measured experimentally by Chang et al.^46^. In both cases, the characteristic current fluctuations of parallel-connected packs are observed. It is the properties of these fluctuations that are examined in this work for the purpose of fault detection.

**Fig. 3:**
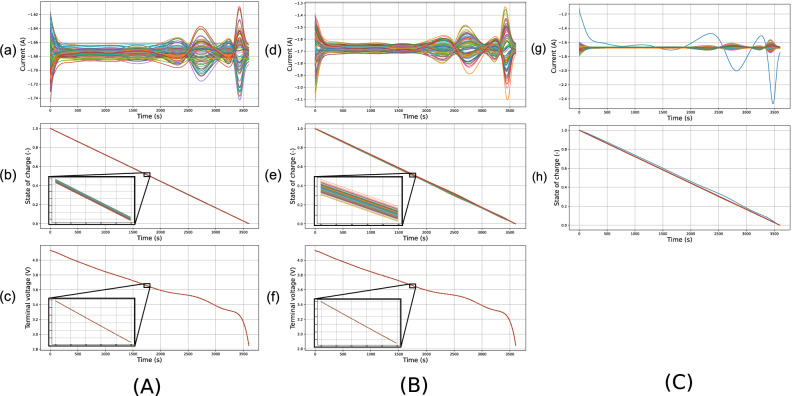
Simulated currents, state-of-charges, and voltages for 74 parallel-connected cells during a 1C discharge. **A** Fresh Tesla Model S module: currents (**a**), states of charge (**b**), and voltages (**c**). Each line corresponds to a cell. **B** Aged Tesla Model S module: currents (**d**), states of charge (**e**), and voltages (**f**). Each line corresponds to a cell. **C** Pack with one faulty cell: currents (**g**) and states of charge (**h**), with the solid cyan line indicating the defective cell among 74 cells.

The architecture of a Tesla Model S battery was used as a reference for a large parallel-connected pack. This pack has a 74p96s configuration, but a single module (74p1s configuration) was simulated here. Because all modules are subjected to the same pack current, they all behave similarly, hence our focus on the simulation of a single module. The 74 cells of the module were assigned parameters according to the normal distribution defined previously (Tables 1 and  2), and the ODEs of the pack model were solved to obtain the terminal voltage, state-of-charge, and current flowing through each cell in parallel during a full discharge. The results of this pack-level simulation are shown in Fig. 3. The observed current fluctuations typically have an amplitude of a few hundredths of an ampere and are therefore detectable with shunt current sensors that typically have tolerances ranging from 0.1 to 1% and can be connected in series with the cells^14^– although, in practice, cost reasons make such mass sensing impractical. The highest current deviations from the mean are located at the beginning and end of the discharge.

At the start of the simulation, the immediate distribution of currents across the pack is due to differences in series resistance from cell-to-cell causing different currents to flow through the cells such that they have the same terminal voltage. Because of this initial current distribution, the cells discharge at different rates. The values of the cell OCVs will then also vary from cell-to-cell, since the OCV is a function of the state-of-charge, causing the currents within the pack to rebalance themselves to enforce Kirchhoff’s voltage laws. This rebalancing mechanism is the reason why the fluctuations are more visible at the end of the discharge where the gradient of the OCV with respect to SoC changes is steep. It should also be noted from Fig. 3 that the SoCs varies slightly between the cells and that the terminal voltage is the same throughout the parallel pack, which is consistent with the Kirchhoff law of Equation 2.

It should be recalled that the cells modelled during this study were considered to be fresh and from the same batch, implying little cell-to-cell variability (except for the faulty cells used for the fault detection algorithm that were deliberately selected to have a high variation so as to represent a fault). For comparison, a simulation of the same pack but with the fresh cells replaced with aged ones was run, as shown in Fig. 3. To characterise the aged cells, the value of the standard deviations of the parameters was multiplied by five with respect to the values chosen previously (see Table 2). The current variations follow a similar shape to the fresh pack simulation of Fig. 3, with one significant difference; the amplitude of the variations is one order of magnitude higher. This increased variation in the currents is responsible for the significantly higher SoC deviations of the aged pack when compared to the healthy pack results in Fig. 3, which are amplified further by the increased variation in the capacitance of each cell. These capacitance differences also cause a larger shift in the current fluctuations over time; the current deviations of the cells with the highest capacitances have a slight delay compared to others, which is particularly noticeable at around 2800 s in the simulation. The simulations suggest that a degradation fault would be more easily identifiable in aged packs compared to fresh ones, as the current fluctuations would be greater. In the remainder of this paper, the focus will be on the design of fault-detection algorithms for the pack described above.

The general form of the cell-level voltage equations of Eq. (1) allows the equation for the branch currents given in Equation 3 to be applied to a broader class of models than just the circuit model discussed above. In particular, it can be applied to DFN-style electrochemical Li-ion battery models^47^ with double layer effects included^48,49^—a benchmark model for Li-ion batteries. Electrochemical battery models are, generally, more complex than circuit models and this added complexity introduces challenges when using them within pack models, as observed in the recent work of Reniers and Howey^50^. In that work, a pack-level model was developed for a 1 MWh grid battery system containing 18,900 cells; simulations then cycled the system for 10 years. The cell-level model of that study was the single particle model (SPM), and although this is one of the simplest forms of battery electrochemical models, the analysis highlighted the difficulty of resolving Kirchhoff’s laws for parallel connections of SPMs. Specifically, due to the SPM nonlinear series resistance, the parallel Kirchhoff laws had to be resolved using an approach based upon a PID controller, which introduced some errors.

By contrast, it is shown in the Supplementary Information (SI1 giving the mathematical analysis and SI2 defining the DFN model variables and parameters) that resolving parallel connections of DFN models is intuitive and possibly easier to implement than for the SPM. Even though the DFN model is more complex than the SPM, this result shows how computing the branch currents is easier. As detailed in SI1, this is obtained by expressing the DFN model voltage within the anode, cathode, and separator in the form of Eq. (1) with

$$r={R}_{{{{\rm{ctc}}}}}+\int_{{\Omega }_{{{{\rm{n}}}}}}\frac{1}{{\sigma }_{{{{\rm{s}}}}}+{\kappa }_{{{{\rm{e}}}}}}\,dx+\int_{{\Omega }_{{{{\rm{p}}}}}}\frac{1}{{\sigma }_{{{{\rm{s}}}}}+{\kappa }_{{{{\rm{e}}}}}}\,dx+\int_{{\Omega }_{{{{\rm{s}}}}}}\frac{1}{{\kappa }_{{{{\rm{e}}}}}}\,dx,$$

and

$$h({x}_{k}(t))= 	 {\phi }_{{{{\rm{dl}}}}}(L,t)-{\phi }_{{{{\rm{dl}}}}}(0,t)+\int_{{\Omega }_{n}}\frac{\omega {\kappa }_{{{{\rm{e}}}}}}{{\sigma }_{{{{\rm{s}}}}}+{\kappa }_{{{{\rm{e}}}}}}\frac{\partial {{{\rm{ln}}}}({c}_{{{{\rm{e}}}}}(x,t))}{\partial x}-\frac{{\sigma }_{{{{\rm{s}}}}}}{{\sigma }_{{{{\rm{s}}}}}+{\kappa }_{{{{\rm{e}}}}}}\frac{\partial {\phi }_{{{{\rm{dl}}}}}(x,t)}{\partial x}\,dx \\ 	 +\int_{{\Omega }_{{{{\rm{p}}}}}}\frac{\omega {\kappa }_{{{{\rm{e}}}}}}{{\sigma }_{{{{\rm{s}}}}}+{\kappa }_{{{{\rm{e}}}}}}\frac{\partial {{{\rm{ln}}}}({c}_{{{{\rm{e}}}}}(x,t))}{\partial x}-\frac{{\sigma }_{{{{\rm{s}}}}}}{{\sigma }_{{{{\rm{s}}}}}+{\kappa }_{{{{\rm{e}}}}}}\frac{\partial {\phi }_{{{{\rm{dl}}}}}(x,t)}{\partial x}\,dx+\int_{{\Omega }_{{{{\rm{s}}}}}}\omega \frac{\partial {{{\rm{ln}}}}({c}_{{{{\rm{e}}}}}(x,t))}{\partial x}\,dx.$$

Crucially, by including double-layer dynamics in the DFN model, the potential *ϕ*_dl_(*x*, *t*) becomes a model state as the double-layer dynamics give it a time derivative. The parallel branch currents can then be computed directly using Equation 3, eliminating the need to solve the algebraic equations of Kirchhoff’s laws when using DFN models. Thus, even though the DFN is a more complex electrochemical model than the SPM, computing the branch currents for parallel connections is intuitive and can be readily implemented, with the branch currents expressed directly as a function of the model states and the applied current.

To verify that Kirchhoff’s laws had been satisfied with this DFN pack model, simulations of two parallel-connected DFN electrochemical models were conducted. The parameters of these simulations are given in SI2. The model was formulated numerically by discretising the PDEs using second-order central differences to approximate spatial derivatives, along with averaging of the diffusivity and conductivity functions at the control volume faces, and solved using “ode15s” within MATLAB® 2021b^51^. The parameters for each cell in this pack model are given in Table 1 and Table 2. A constant-current discharge was modelled with a current density of *I*(*t*) = 129.55 A/m^2^. It was assumed that the contact resistance between cells one and two in the simulated parallel pack differed with *R*_ctc_ = 5.7 × 10^−4^ *Ω* for cell one and *R*_ctc_ = 7.7 × 10^−4^ *Ω* for cell two—this setup is referred to as case one. As well as differences between cell contact resistances, two other cases were simulated. Case two additionally has the anode reaction rate coefficient in cell two being three times that of cell one, while case three has the anode particle radius of cell two being three times greater than in cell one.

**Fig. 4:**
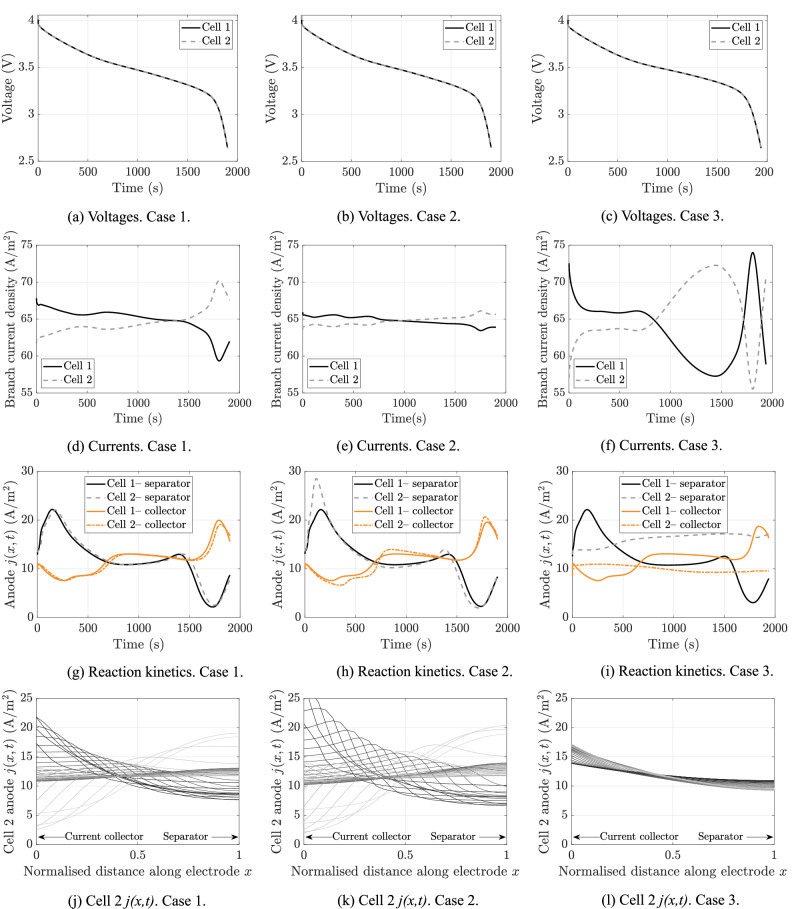
Discharge simulation results of two parallel-connected DFN models using the parameter values in Table 1 and Table 2. Case 1 corresponds to the nominal setup, with *R*_ctc_ = 5.7 × 10^−4^ *Ω* for cell 1 and *R*_ctc_ = 7.7 × 10^−4^ *Ω* for cell 2. Case 2 additionally has an anode reaction rate coefficient *k* three times greater in cell 2 than cell 1. Case 3 has an anode active particle radius three times greater in cell two than cell one and the fourth row is plotted every 25 seconds, with dark lines corresponding to the start of the simulation and light lines to the end. **a**–**c** are the voltage responses for the three cases, **d**–**f** are the branch current densities, **g**–**i** are the anode reaction rates in time, and **j**–**l** are the distribution of the reaction rates in space across the anode.

The simulation results for the three cases discussed above are shown in Fig. 4. In this figure, the first row describes the pack voltages, the second row is the branch currents, the third row is the evolution of the cathode reaction rate kinetics at both the current collector and the separator boundaries, and the fourth rows corresponds to the evolution of the spatial distribution of the reaction kinetics through the thickness of the anode. Each line in the fourth row figures corresponds to a snapshot of *j*(*x*, *t*) in the anode taken every 25 s during simulation, with darker lines corresponding to the start of the simulation and lighter lines corresponding to the end. The three columns in the figure corresponds to the three different cases of the cell parameters being simulated.

From the current and voltage plots of Fig. 4, it can be observed that Kirchhoff’s laws are satisfied; cell voltages are equal and the current splits between the two cells and constantly rebalances itself during the discharge. The three different cases were found to lead to three different responses, highlighting the degree to which manufacturing variability of cells (and hence, variation in their electrochemical parameters) can affect pack performance. The final two rows in the figure illustrate how variations in cell parameters can lead to variations in the electrochemical response. These variations may impact the pack response in the long-term, especially cell degradation rates^52^.

The generalisation to parallel-connected DFN models discussed above shows how the modelling framework of Equation 2 can be applied to more complex models, beyond simple electrical circuits. The framework can also be generalised to time-varying current profiles, such as the drive cycles considered in ref. ^44^, and is not restricted to the constant-current profiles used here for fault diagnosis. With this approach, detailed simulations of pack electrochemical response can be undertaken. However, due to the significant computational complexity and lack of effective parameter estimation methods for DFN models, it was decided to base the fault detection algorithm here on parameterised circuit models for 74p96s Tesla Model S modules. Once the computational and parameterisation issues of DFN models are resolved, the expression for the branch currents stated in Equation 3 can then be used for pack-level diagnostics with more complex models, such as designing advanced fault-tolerant algorithms.

### Fault detection

To implement the cell fault-detection algorithm, a cell fault must first be introduced. Here, the focus is on understanding faults caused by accelerated degradation. Amongst other phenomena, as a cell ages its resistance increases—especially at end-of-life^53^. Within a pack, some cells will reach this stage sooner than others, leading to potential accelerated degradation faults. For this reason, the resistances of the cells within a pack can be regarded as useful indicators of cell faults, as they can significantly influence the pack dynamics^52^. We therefore consider the detection of faults caused by an abnormal increase in the ohmic resistance of a cell due to accelerated degradation^54^. According to the cell model described in Fig. 1, a cell whose series resistance *r* follows a normal distribution based upon the values given in Tables 1 and 2 will be defined as healthy. A faulty cell will be defined by an abnormally high resistance *r* (at least 5 standard deviations above the mean). Experimental studies^55^ have shown that series resistances of cells within packs can vary by several dozen percent during ageing. For this reason, the following analysis will consider the case when the faulty cell has an increased resistance ranging from 10 to 100%.

To evaluate the impact of cell faults on the current distributions across parallel-connected packs, simulations of both a healthy pack (see Fig. 3) and one with a fault were carried out. The pack containing the fault was similar in all aspects to the healthy pack but one of the cells had an increased series resistance, *r* = 1.5 × *μ*_*r*_ = 28.5 m*Ω*. The results are shown in Fig. 3. There is a clear difference compared to the healthy pack in Fig. 3: the faulty cell exhibits a discharge current at *t* = 0 with a low absolute value due to its resistance fault. Consequently, the current variations experienced by this cell are extreme in amplitude. However, in general, the currents flowing through the non-faulty cells in this aged pack all behave similarly to those obtained for a healthy pack. This similarity between the currents in fresh and aged packs introduces a challenge for fault detection when not all the currents are being monitored.

A fault detection algorithm based upon a SVM classifier was trained on the dataset to perform binary classification between healthy and faulty parallel packs based upon the simulated current distributions, with details of this algorithm given in the Methods section. Features for the SVM fault-detection algorithm were extracted from the dataset samples in order to discriminate the healthy packs from the faulty packs. The performance of the classifier was first verified for detecting faulty packs from all the simulated currents as inputs. Using simple features based on the deviation of currents from the mean, it was expected that faulty packs could be detected because they present an abnormal current signal (see fault detection section). Once trained, the classifier was able to classify the 200 samples of the training set without making any classification errors, thus achieving a 100% accuracy score. The algorithm thus performed successfully when all the currents in the pack were being monitored.

**Fig. 5:**
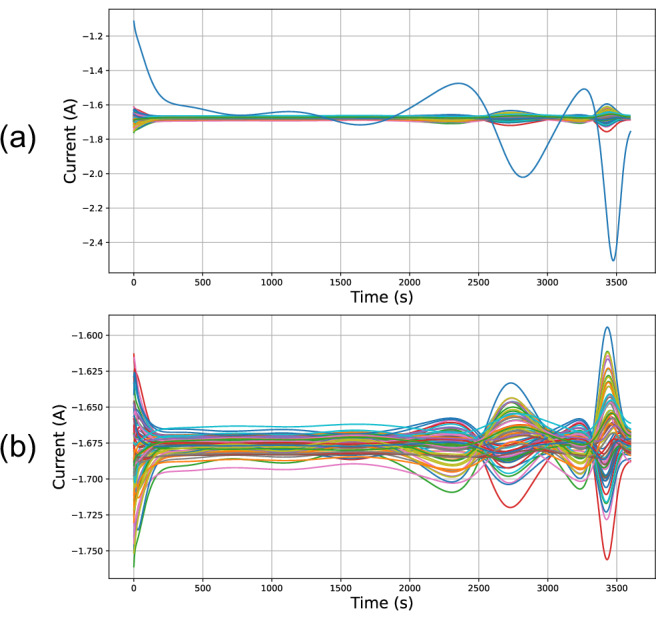
The impact of cell faults on pack-level current distributions. Comparison between the current distributions of a faulty pack (**a**) and the same pack without the faulty cell current (**b**) during a 1C discharge. Each line corresponds to a cell, with the solid cyan line in (**a**) corresponding to the defective cell.

However, in practice, only a limited number of branch currents are typically measured with large parallel-connected packs due to the increased costs of the sensors and added system complexity. To make the proposed algorithm more applicable the fault detection algorithm for the sparse sensing problem is now considered—i.e., when only a fraction of the number of branch currents are measured. With sparse sensing, fault detection becomes significantly harder, as it is challenging to infer the impact of a faulty cell on the rest of the pack when only measuring the healthy-cell currents, as seen in Fig. 5. For this analysis, it was assumed that only *N*_*s*_ current sensors were deployed in the pack and the current from the faulty cell was not measured by these sensors.

Features for the developed SVM fault-detection algorithm were extracted from the dataset samples in order to discriminate the healthy packs from the faulty packs. The extracted features described in the Methods section were then used and an ablation study was performed to characterise their impact on the classification, where the impact of each feature on the classifier was quantified. After empirically testing and selecting features, it was noticed that the most effective descriptors were formed from the local minima and maxima of the current signals. The classification was performed for a range of numbers of sensors *N*_*s*_ in the parallel pack. The classifier was first trained and tested for *N*_*s*_ = 73, i.e. all cell currents available except the faulty cell current. The confusion matrix of the corresponding predictions is given in Table 3a. The same procedure was then applied for *N*_*s*_ = 20, i.e., sensors measuring only a subset of the 74 cells, with results given in Table 3b.

**Table 3:** Confusion matrices for two sensor configurations

		Prediction		
		Faulty pack	Healthy pack	Total
Truth	Faulty pack	81	17	98
	Healthy pack	5	97	102
	Total	86	114	200
(a)Confusion matrix of SVM classifier with *N*_*s*_ = 73 (i.e. all but the faulty parallel branch currents are measured).				

		Prediction		
		Faulty pack	Healthy pack	Total
Truth	Faulty pack	76	27	103
	Healthy pack	7	90	97
	Total	83	117	200
(b) Confusion matrix of SVM classifier for *N*_*s*_ = 20.				

In practice, these measurements may not be available because current sensors are usually not added to every branch of a parallel-connected pack. In fact, often only a few sensors are used due to added costs and additional compute power required from the BMS to process the data. For these reasons, a fault-detection algorithm must be robust in the sense that it should still function even when the current of the parallel branch is not measured. The results in the following section address this issue.

**Fig. 6:**
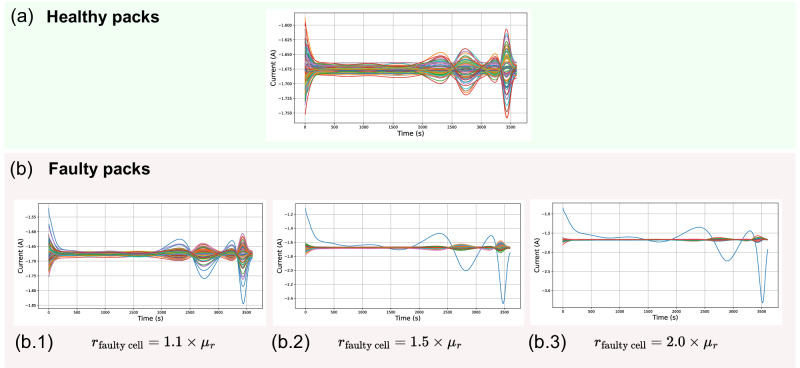
Change in pack-level current distributions as the faulty cell’s resistance increases. Simulations of Tesla Model S battery pack module when all cells are healthy (**a**) and when one of the cells is faulty (**b**). The value of the faulty cell resistance (*r*_faultycell_) varies between cases b.1, b.2 and b.3. It is defined as a multiple of pack mean resistance *μ*_*r*_, and significantly impacts pack current distribution and hence the performance of the fault classification algorithm.

**Fig. 7:**
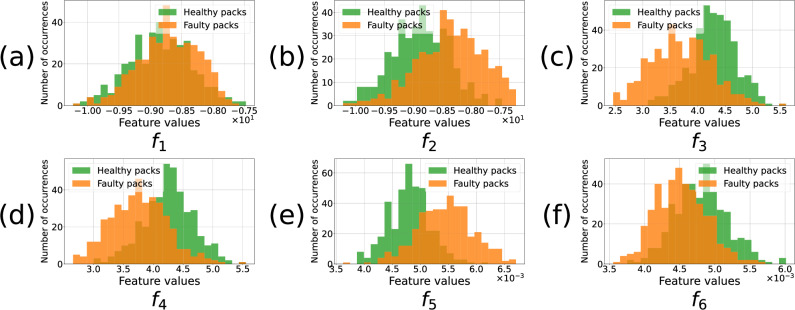
Histograms of feature for healthy and faulty packs of the simulated dataset. **a** gives the distribution of feature *f*_1_ defined in (11), and similarly **b** in (12), **c** in (13), **d** in (14), **e** in (15), and **f** in (16).

The features were then trained on the dataset generated by this model, whose structure is shown in Fig. 6. The profile of the current distributions simulated within the dataset can be seen in this figure, showing that faulty packs can be easily distinguished from healthy packs using measurements of the faulty cell current (with the exception of the case *r*_faultycell_ = 1.1 × *μ*_*r*_). This again highlights the increased difficulty in designing the fault-detection algorithm for the sparse sensing problem. After calculating the features for all samples in the training and testing set, their histograms were analysed. These were calculated for *N*_*s*_ = 73 (i.e. the current signal from the faulty cell was removed in faulty packs, while a random current was removed for healthy packs). The feature values for the healthy and faulty packs were separated, with the resulting histograms compared in Fig. 7. The observed distributions for these features differ between healthy and faulty packs, except for *f*_1_.

The histograms reveal that the extrema reached by the currents, which form the basis of the six identified features *f*_1_ to *f*_6_, allow one to partially discriminate the two classes. This is the case even though it appears that the current distributions of the cells in a healthy pack and the healthy cells in a faulty pack are similar (from Fig. 3 and the fault-detection algorithm results). It is this discrimination that allows the SVM algorithm to detect faults even when only limited sensor information is used. After empirically testing and selecting different features, it was also noticed that the most effective descriptors were formed from the local minima and maxima of the current signals. Finally, it is noted that as the number of removed current sensors increases, the distributions of the histograms of the two pack classes (healthy and faulty) overlap—resulting in reduced classification performance.

**Fig. 8:**
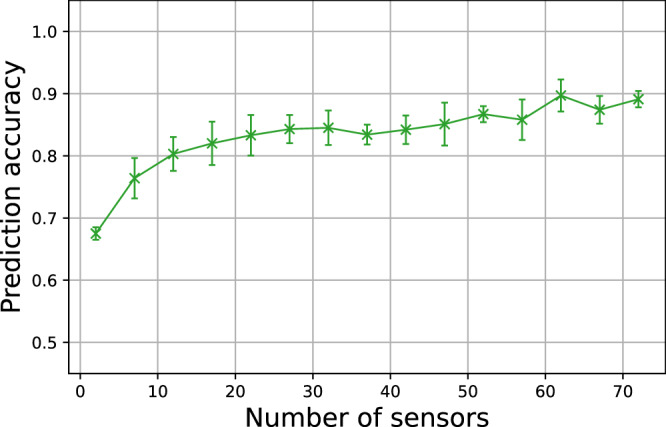
Accuracy score of fault-detection classifier (SVM) as a function of *N*_*s*_ (the number of current sensors within the 74p1s parallel pack). For each number of sensor plotted, 50 classifiers were trained, with the crosses representing the median accuracy and the bars representing the standard deviation of the accuracy across the tests.

Finally, the SVM classifier was trained and tested for a larger number of *N*_*s*_ values, between 2 and 72, and the model accuracy evaluated for each trial. The results are shown in Fig. 8 and are a key contribution of this paper. This figure compares the accuracy of the proposed faulty pack detection algorithm as a function of the number of measured current branches *N*_*s*_. A plateau can be seen in the prediction accuracy of the figure at around 20 sensors out of 74, continuing to around 50 sensors. This suggests that the optimal detection configuration for the Tesla Module S module, in terms of precision and sensing cost, is around 20 sensors. As the number of deployed sensors decreases, the accuracy score decreases: for 7 sensors (10% of the cells in the pack), an accuracy of 76% was obtained, and for 2 sensors only, it dropped to 67%.

Table 3b details the predictions made by the algorithm with only 20 sensors—the results contain few false positives, and a larger quantity of false negatives. Since an accelerated cell degradation fault in a pack is a relatively infrequent phenomenon, it is important that there are few false positives. However, false negatives can be problematic and it can be seen that this is the main difference compared to the *N*_*s*_ = 73 case discussed in Table 3a, where there are fewer false negatives. Considering the plot shown in Fig. 8, and the statistical nature of the extracted features used by the detection method, it appears that this method is applicable for a parallel pack made of a large number of cells (*N*_*c*_, total number of cells in pack), such as the Tesla Model S module. However, for pack configurations where only a few cells are connected in parallel, a method that uses only a small fraction of the total number of cells in the module may prove difficult to implement.

The accuracy of the proposed SVM fault classifier was also evaluated against a recurrent neural network (RNN), since RNNs are widely used in many battery system algorithms, e.g., state-of-charge estimation^56^ because they can implement online diagnosis in an end-to-end manner. Details of the RNN can be found in the Methods section. Training on the raw current data, the RNN had a fault-detection accuracy of 47.0% when *N*_*s*_ = 73 and 45.50% when *N*_*s*_ = 20, lower than the SVM. Whilst other neural network architectures may show higher accuracies, the results indicate that simple SVM classifiers can perform well for this task when compared against more complex deep learning methods. The simplicity of the SVM also brings advantages for practical deployment, both in terms of explainability of features but also compact size. However, deep learning approaches, such as the RNN considered here, will likely outperform SVMs for more diverse and complex usage profiles than the constant discharge currents of this paper. With rapidly changing use profiles, such as drive cycles, the SVM features may not be appropriate as it would be challenging to disentangle the fluctuations caused by the use profile and those caused by the imbalances across the parallel pack. Future work and experimental data will be required to validate this.

Several model parameters strongly influence the dynamic response of the current distributions across a parallel-connected pack and, consequently, the performance of the detection algorithm. For example, it was observed that the points where the fluctuations in the current distributions were largest, during both discharging and charging, correlated with the points where the slope of the OCV curves were steepest. The shape of the OCV curve (which is different for each cell chemistry) therefore strongly impacts the ability of the algorithm to detect cell faults. Additionally, it is noted that the the pack ageing will influence the detection algorithm—the variation between cell parameters may grow, which could lead to greater current deviations and potentially improve the accuracy of the detection algorithm. It should also be noted that the obtained accuracy of 82.8% for *N*_*s*_ = 20 (i.e. sensors placed on about 27% of the cells in the pack) assumes that no sensor is ever placed on the faulty cell. Since this should happen in this configuration in 27% of cases and the fault is systematically detected in this case, we can assume a higher accuracy in the general case.

## Experimental validation

**Fig. 9:**
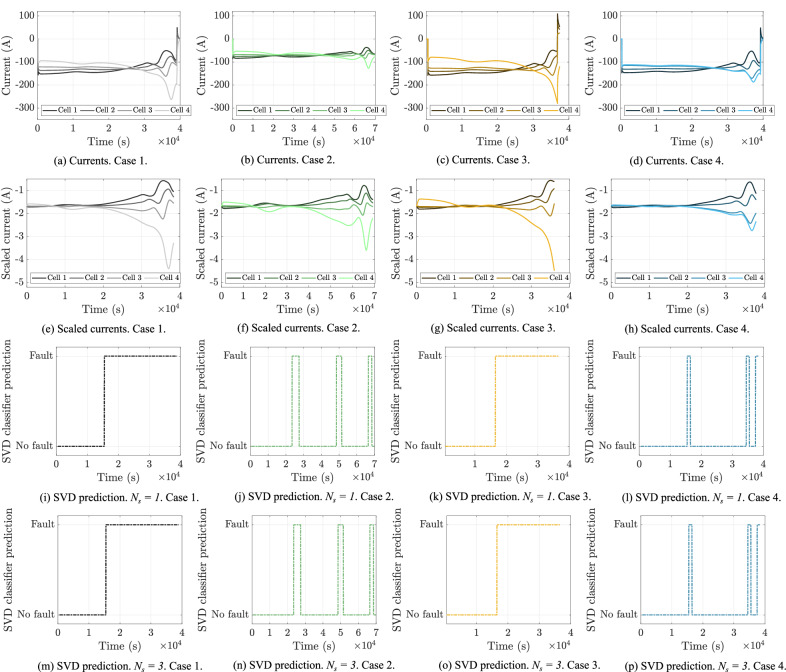
Application of SVD classifier to detect faults in experimental pack data with four RJ-LFP72174204-280 cells connected in parallel. As detailed in Methods Section, four fault cases were considered, with Cases 1–3 being faulty and Case 4 fault free. **a**–**d** show experimental current data, **e**–**h** show scaled currents, **i**–**l** show fault predictions with *N*_*s*_ = 1 and **m**–**p** show fault prediction with *N*_*s*_ = 3.

To evaluate the practical applicability of the proposed approach, the SVD fault prediction classifier trained on 74p1s pack simulations with NCR 18650B cells was then applied to experimental testing data. Details of the experimental setup can be found in the Methods section. Figure 9a–d show the recorded current data of the four cells in the packs for the four cases. In order for the experimental current data to be in the same format as that of the SVD’s training data (e.g., that shown in Fig. 6), the data was scaled according to the approach detailed in the Methods Section, with the normalised currents shown in Fig. 9e–h. Feeding this scaled experimental data into the SVD classifier trained on the 74p1s pack NCR 18650B cell simulation data gave the predictions of: (i) Fig. 9i–l with *N*_*s*_ = 1, and (ii) Fig. 9m–p with *N*_*s*_ = 3. In general, the SVD predictor performed relatively well on this experimental dataset. With *N*_*s* _= 1, all the faulty cases were detected, but the healthy pack was also predicted to be faulty; with *N*_*s*_ = 3 (as in with only the faulty cell not being sensed), the SVD predictor detected the faults in Cases 1 and 3 but misclassified Case 2 during most of the discharge and Case 4 at some times. In general, these results highlight the transferability of machine learning algorithms for battery systems trained on simulation data to experimental data, with the SVD classifier in Fig. 9 performing satisfactorily even though it was trained on 74p1s pack simulation data with NCR 18650B cells and tested on experimental pack data of four RJ-LFP72174204-280 cells.

## Conclusions

A method to detect cell faults in large parallel connected battery packs that combines model-based prediction with machine learning classification was presented. An equivalent circuit model of a parallel battery pack was developed to simulate the behaviour of an EV battery pack, specifically a Tesla Model S module. Current distributions within the parallel connected pack were studied in detail and an expression for the branch currents as a function of the model’s states and the pack’s applied current was derived. This expression for the parallel-pack branch currents was shown to generalise to more complex battery models, including the DFN electrochemical model. The branch currents of the parallel connected were used within an SVM-based algorithm to detect faulty cells in the pack to improve its safety. The proposed fault-detection algorithm was shown to perform well, achieving an accuracy of 83% when using only 20 current sensors per module of 74 cells in parallel. To assess its applicability in practice, the algorithm trained on simulation data was then testing on experimental pack data and performed well, with the results shown in Fig. 9. These results demonstrate the potential of detecting cell degradation faults with sparse sensing on large-scale parallel battery packs. By combining algorithms with current sensing data in this way, these results can be used to improve the safety of a battery pack.

One limitation of this paper is the focus on faults from resistance increases in one of the pack’s cells. A wider study including faults from both capacitance losses and impedance growth would result in a more comprehensive detection algorithm. The motivation to focus on resistance faults here was: (i) they could be replicated in experiments (see e.g. Fig. 9), (ii) these faults could occur quite suddenly, such as a tab becoming loose or lithium plating when fast charging in sub-zero temperatures, whereas slower degradation effects, such as capacitance losses from SEI layer growth, may be corrected by the averaging effect of the parallel connections, (iii) the higher resistances from the faults could lead to higher heat generation rates and so impact the pack’s safety through thermal runaway. Generalising the results of this paper to the more diverse class of faults and current profiles found in practice is an important direction for future research in this area. For that generalisation, a rich-dataset containing a wide class of faults in practical battery systems would be required which would be challenging to achieve at scale.

As well as generalising the results to a more diverse class of faults, the authors are also comparing the efficacy of the proposed SVM algorithm with other approaches based upon deep learning, such as convolutional neural networks. As these algorithms have delivered strong results in other classification tasks, it is expected that they may also perform well for the problem considered here of detecting faults in large parallel connected packs. However, neural networks have limitations which may affect their performance in practice, notably, their lack of robustness may impact the extent to which engineers trust them for monitoring large and expensive packs, where safety issues need to be understood and failures explained.

## Methods

### Cell model

**Fig. 10:**
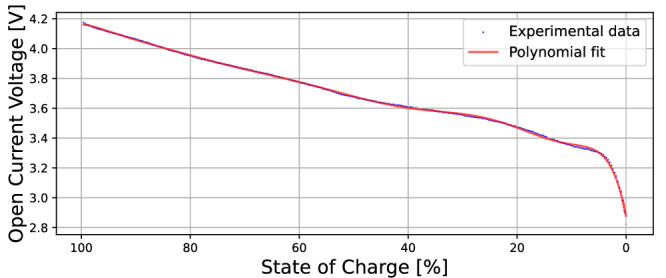
The open circuit voltage curve used for the Panasonic NCR 18650B cells. Comparison between the digitised OCV curve from ref. ^58^ (blue) and its polynomial interpolation (red).

The pack model is based on the equivalent circuit model of a single cell and the connection of multiple cells in parallel. The cell model used is the Thevenin model shown in Fig. 1 which has been widely used due to its simplicity combined with its high accuracy^57^. In this model, the dynamics for each cell *k* ∈ {1,  2,  …,  *n*} are characterised by its series resistance *r*_*k*_, RC-pair resistance *R*_1,*k*_, RC-pair capacitance *C*_1,*k*_ and capacity *Q*_*k*_. The subscript *k* is dropped from these parameter labels when referring to them in a general context, for example in Table 1 where their mean values are stated. The battery pack of a Tesla Model S was modelled and so the modelled pack’s cells were chosen to be Panasonic NCR 18650B cells. The corresponding model parameters are given in Table 1 whilst the OCV curve was obtained from experimental data^58^ and fitted using a polynomial approximation (see Fig. 10). The branch current *i*_*k*_(*t*) flowing through cell *k* was considered to be positive when charging and negative when discharging.

Within the pack model, the dynamics for cell *k* ∈ {1,  2,  …,  *n*} were described by the simple equivalent circuit model

4a $$\left[\begin{array}{c}{\dot{z}}_{k}(t)\\ {\dot{V}}_{1,k}(t)\end{array}\right]=\left[\begin{array}{cc}0&0\\ 0&-\frac{1}{{R}_{k}{C}_{k}}\end{array}\right]\left[\begin{array}{c}{z}_{k}(t)\\ {V}_{1,k}(t)\end{array}\right]+\left[\begin{array}{c}1/{Q}_{k}\\ 1/{C}_{1,k}\end{array}\right]{i}_{k}(t),$$

4b $$V(t)=OCV({z}_{k}(t))+{V}_{1,k}(t)+{r}_{k}i(t),$$

where, for cell *k* ∈ {1,  2,  …,  *n*}, *V*_1,*k*_(*t*) is the voltage drop across the RC-pair of Fig. 1, *z*_*k*_(*t*) is the state-of-charge, *V*(*t*) is the voltage and *OCV*(*z*(*t*)) is the open circuit voltage.

### Parallel pack model: conversion of DAEs into ODEs

The parallel connection of *n* cells, as described in Fig. 1, was then modelled in accordance with Kirchhoff’s laws. For parallel connections, these laws impose that the voltages *V*(*t*) across each of the cells are the same and that the sum of the cell branch currents must equal the total current applied to the pack.

Defining *x*_*k*_(*t*) as the state vector of cell *k*, which for the circuit model is ${x}_{k}(t)={[{z}_{k}(t),{V}_{1,k}(t)]}^{\top }$, and *i*_*k*_(*t*) as the current flowing through cell *k* in response to the pack current *I*(*t*), then, for *j*, *k* ∈ {1,  2, …,  *n*}, Kirchhoff’s laws for parallel connected packs can be written as

5a $$OCV({z}_{j}(t))+{V}_{1,j}(t)+{r}_{j}{i}_{j}(t)=OCV({z}_{k}(t))+{V}_{1,k}(t)+{r}_{k}{i}_{k}(t),$$

5b $${\sum}_{k=1}^{n}{i}_{k}(t)=I(t).$$

Combining Equation 4 and Equation 2 gives the system of DAEs of the parallel pack model. The use of DAE models is, however, often undesired because they can be significantly more challenging to simulate and analyse than those described by ODEs. In the following analysis, a method to translate this DAE model of the parallel pack into an ODE one is described. The first step of this conversion is to express Kirchhoff’s laws as

6a $$V(t)={r}_{k}{i}_{k}(t)+{V}_{1,k}(t)+OCV({z}_{k}(t))$$

6b $$={r}_{k}{i}_{k}(t)+h({x}_{k}(t)),\forall k\in \{1,\,2,\ldots ,\,n\},$$

where *h*(*x*_*k*_(*t*)) is a function of cell *k*’s state-space. Kirchoff’s laws can then alternatively be written as

7a $${r}_{1}{i}_{1}(t)-{r}_{2}{i}_{2}(t)=\Delta {h}_{21}(t),$$

7b $$\begin{array}{l}\vdots \\ {r}_{1}{i}_{1}(t)-{r}_{n}{i}_{n}(t)=\Delta {h}_{n1}(t),\end{array}$$

7c $${i}_{1}(t)+{i}_{2}(t)+\cdots +{i}_{n}(t)=I(t),$$

where Δ*h*_*jk*_(*t*) = *h*(*x*_*j*_(*t*)) − *h*(*x*_*k*_(*t*)). With this formulation, each of the branch currents *i*_*k*_(*t*) can be expressed in terms of the first one, as in

8a $${i}_{2}(t)=\frac{1}{{r}_{2}}\left({r}_{1}{i}_{1}(t)-\Delta {h}_{21}(t)\right),$$

8b $$\begin{array}{l}\vdots \\ {i}_{n}(t)=\frac{1}{{r}_{n}}\left({r}_{1}{i}_{1}(t)-\Delta {h}_{n1}(t)\right).\end{array}$$

Substituting these expressions back into Eq. (7c) gives

9a $$I(t)={i}_{1}(t)+{i}_{2}(t)+\cdots +{i}_{n}(t),$$

9b $$={i}_{1}(t)+{\sum}_{k=2}^{n}\frac{1}{{r}_{k}}\left({r}_{1}{i}_{1}(t)-\Delta {h}_{k1}(t)\right),$$

9c $$=\left(1+{r}_{1}{\sum}_{k=2}^{n}\frac{1}{{r}_{k}}\right){i}_{1}(t)-{\sum}_{k=2}^{n}\frac{\Delta {h}_{k1}(t)}{{r}_{k}}.$$

The first branch current *i*_1_(*t*) can then be written exclusively in terms of the model’s state and the applied current via

10 $${i}_{1}(t)={\left({r}_{1}{\sum}_{k = 1}^{n}\frac{1}{{r}_{k}}\right)}^{-1}\left({\sum}_{k=2}^{n}\frac{\Delta {h}_{k1}(t)}{{r}_{k}}+I(t)\right).$$

Using Equation 3, the other cell currents *i*_2_, … , *i*_*n*_ can then also be written in terms of the model states and the applied current. This process allows the parallel pack model to be described by an ODE instead of a DAE, which greatly simplifies the analysis^30^. The ODE model equations were then solved numerically using the integrate.odeint solver from the scipy package in Python.

### Data processing pipeline

The faulty cell detection method is implemented through the following data processing pipeline:

1. Data Simulation. A large quantity of battery pack discharges, including both healthy and faulty packs, is simulated using the pack model developed around Equation 2 to generate a substantial number of current distributions.
2. Data Pre-processing. The simulated data is pre-processed to emulate the acquisition process of the current sensors used in practice. The data is then divided into a training set and a testing set.
3. Feature Extraction. Heuristic features are constructed from the pre-processed data to facilitate the classification of healthy and faulty battery packs.
4. Binary classification. A SVM is trained on the extracted features to perform binary classification, distinguishing between healthy and faulty battery packs.

Each pack was characterised by a set of 74 time signals (corresponding to the 74 cells in the Tesla Model S module). The generated dataset contained 1000 samples consisting of the simulated currents of the parallel pack. Each sample is represented as a matrix of size $74\times {t}_{\max }$ with ${t}_{\max }\approx$ 3600 s, being the duration of the pack discharge. A total of 500 healthy and 500 faulty packs were simulated. The healthy packs are assigned parameters following the normal distribution of the model for all 74 cells, while the faulty packs contain a faulty cell whose resistance *r*_faultycell_ has been set to be abnormally high (as defined at the beginning of the fault detection section). Different degrees of fault are considered during the simulations. Among the 500 simulated faulty packs, 50 are assigned the faulty cell resistance value *r*_faultycell_ = 1.1 × *μ*_*r*_, 50 are assigned the value *r*_faultycell_ = 1.2 × *μ*_*r*_, and so forth until *r*_faultycell_ = 2.0 × *μ*_*r*_.

### Data pre-processing

The first step of the data pre-processing phase involves removing a number of current sensors from the samples of the data-set. By removing the current data of the faulty cell, the performance of the fault-detection algorithm was made more robust and more representative of packs deployed in practice, as costs may limit the number of branch currents being monitored. In fact, it was found to be straightforward to distinguish a healthy pack from a faulty one when all the current distributions were measured (i.e. when all the currents flowing through the cells were known, including that of the faulty cell). By deliberately removing the data generated by the faulty cell from the algorithm’s input, the fault detection problem becomes non-trivial. If the current from the faulty cell is not measured, the behaviour of the current distributions appears to be similar to that of a healthy pack, can be seen from the results of Fig. 6. The method presented here thus aimed to perform faulty pack detection using only a fraction of the total potential current sensors. To do this, first, a given number of current signals, *N* = *N*_*c*_ − *N*_*s*_≥ 1 where *N*_*c*_ is the total number of cells in the parallel pack and *N*_*s*_ is the number of sensors that are ultimately used, were removed from each sample of the dataset. This is equivalent to placing sensors on only a few of the 74 branches of the Tesla Model S’s parallel pack module. For the faulty packs, the current sensor of the faulty branch is removed and the other removed current sensors are randomly selected.

The second step of the data pre-processing stage consisted in adding noise to the data and then filtering it. This noise was added to the model simulation data to make it more realistic with respect to the current sensor data measured from experiments. Given that the shunt resistors used for current sensing can generate a measurement error of approximately 0.1%^14^, a Gaussian noise with an amplitude of  ±0.1% of the current signals was used. To be conservative, the standard deviation of the Gaussian noise distribution for each current signal was set to 0.05% of the mean value of the current during discharge. All the signals were then filtered by a low-pass Butterworth filter of order 5, and critical frequency *f*_*c*_ = 0.005 Hz (the sampling frequency being *f*_*s*_ = 1 Hz). These operations resulted in slightly distorted signals compared to the original data.

The third and last step of the data pre-processing stage is the train-test splitting of the data. The dataset was split into 80:20 proportions for the training and the testing set respectively; a 5-fold selection is applied for this purpose. These two sets contained samples consisting of matrices of size ${N}_{s}\times {t}_{\max }$. Features are then extracted from these samples and, after the feature transformation has been applied, each sample consists of a vector of length ${N}_{{{{\rm{features}}}}}$. The standard technique of training an SVM classifier^59^ was then applied to the datasets.

### Feature extraction

Six features, labelled as *f*_1_ to *f*_6_, were considered. Each sample consists of a matrix containing in each of its rows a branch current time-series. To be specific about individual time-series within those for the whole pack, the row index *p* is used to denote a given cell current within the parallel pack, and *t* will refer to the time index of the signal. The total number of cells is *N*_*c*_ and the duration of the time-series is ${t}_{\max }$, with a time step Δ*t* = 1s between each sample *t*. A given sample of the dataset is denoted by *S* and the value of the current in cell *p* at time *t* within the signal is *S*[*p*, *t*].

1. Feature 1 is the average sum of local maxima over the cell currents across the whole discharge. Writing the local current maximum on a given time interval within a cell as ${\max }_{t\in I}S[p,t]$, and the k^th^ local maximum current encountered during its discharge (out of *N* local maxima) as ${\left({\max }_{t}S[p,t]\right)}_{k}$, the feature was then defined as:
  11 $${f}_{1}=\frac{1}{{N}_{c}}{\sum}_{p=1}^{{N}_{c}}{\sum}_{k=1}^{N}{\left({\max }_{t}S[p,t]\right)}_{k}.$$
2. Feature 2 characterised the local current minima instead of the local maxima:
  12 $${f}_{2}=\frac{1}{{N}_{c}}{\sum}_{p=1}^{{N}_{c}}{\sum}_{k=1}^{N}{\left({\min }_{t}S[p,t]\right)}_{k}.$$
3. Feature 3 uses the standard deviation instead of a mean:
  13 $${f}_{3}=\sqrt{\frac{1}{{N}_{c}}{\sum }_{p=1}^{{N}_{c}}\left[{\left[{\sum }_{k = 1}^{N}{\left(\mathop{\max }_{t}S[p,t]\right)}_{k}-{f}_{1}\right]}^{2}\right]}.$$
4. Feature 4 was defined similarly to *f*_3_ but used the local minima:
  14 $${f}_{4}=\sqrt{\frac{1}{{N}_{c}}{\sum }_{p=1}^{{N}_{c}}\left[{\left[{\sum }_{k = 1}^{N}{\left(\mathop{\min }_{t}S[p,t]\right)}_{k}-{f}_{3}\right]}^{2}\right]}.$$
5. Feature 5 was based on the standard deviation of all local maxima within the signals (rather than taking their sum as in *f*_3_) for a given *p*:
  $$\left\{{\max }_{t}S[p,t]\right\}=\left\{{\left({\max }_{t}S[p,t]\right)}_{0},\,\ldots \,,\,{\left({\max }_{t}S[p,t]\right)}_{N}\right\}.$$
  We define
  $$\max S=\left\{{\max }_{t}S[1,t],\,...\,,\,{\max }_{t}S[{N}_{c},t]\right\}$$
  and denote its elements as ${\left(\max S\right)}_{i}$ which are numbered from 1 to *N*_*m*_. Then, the feature, corresponding to the standard deviation of all local maxima within a sample was defined
  15 $${f}_{5}=\sqrt{\frac{1}{{N}_{m}}{\sum }_{i = 1}^{{N}_{m}}\left[{\left[{\left(\max S\right)}_{i}-\overline{{\left(\max S\right)}_{i}}\right]}^{2}\right]}.$$
6. The final feature was defined as the standard deviation of all local minima within the signal *S*:
  16 $${f}_{6}=\sqrt{\frac{1}{{N}_{m}}{\sum }_{i = 1}^{{N}_{m}}\left[{\left[{\left(\min S\right)}_{i}-\overline{{\left(\min S\right)}_{i}}\right]}^{2}\right]}.$$

### SVM binary classification

The classification was performed by an SVM classifier^60^ with hyperparameter optimisation using grid search. A 5-fold cross-validation was used to create a validation set from the test set obtained during the train-test split of the pre-processing phase. The hyperparameter grid was:

- C: [0.01, 0.05, 0.1, 0.5, 1, 5, 10],
- *γ*: [10^−5^,  10^−4^,  10^−3^,  0.01,  0.1,  1],
- Kernel: [‘*linear*’, ‘*rbf*’, ‘*poly*’, ‘*sigmoid*’].

Here, C denotes the regularisation parameter, while the kernel function transforms the input data into another, often non-linear and high-dimensional feature space, and *γ* determines the influence of the training samples on the definition of the SVM separation hyperplane. The parameters retained after optimisation were C = 1, kernel = *‘linear*’, and *γ* being used only for the ‘*rbf*’, ‘*poly*’ with ‘*sigmoid*’ kernels^60^. The SVM predictions, *y*, were then obtained by

17 $$y={{{\rm{sign}}}}(wf+b)$$

with *w* being the weights and *b* the bias learned from data.

### Recurrent neural network

The RNN was composed of an input layer, a Long Short Term Memory (LSTM) layer with 100 neurons, a fully connected layer, a softmax layer, and a classification layer. A learning rate of 0.001 was used with a gradient clipping threshold of 1 implemented with the ADAM optimisation algorithm. The inputs to the RNN were the current signals from each measured branch of the parallel pack and its output was a fault diagnosis. The number of epochs was 50 and the mini batch size was 32.

### Experimental setup

Four 280 Ah prismatic LFP cells from RJ Energy (RJ-LFP72174204-280) were connected in parallel and current sensors recorded the branch currents. The sensors were IVT-S shunt sensors manufactured by Isabellenhutte with a  ±1000 A range and shunt resistor values of 20 μΩ. Faults were introduced into Cell 4 of the packs through additional shunt resistors. As detailed in Table 4, four cases were considered with Cases 1–3 being faulty packs and Case 4 being healthy (i.e., without the additional shunt resistor).

**Table 4:** Four cases of packs in the experimental data

	Fault resistance	Discharge C-rate (Current)	Faulty pack
Case 1	125 μ Ω	0.45C (504 A)	*✓*
Case 2	125 μ Ω	0.25C (280 A)	*✓*
Case 3	250 μ Ω	0.45C (504 A)	*✓*
Case 4	0 μ Ω	0.45C (504 A)	✗

The scaled currents, ${\hat{i}}_{k,\ell }(t)$, for cell *k* of Case *ℓ* were calculated from the experimental current data,*i*_*k*,*ℓ*_(*t*), using

18 $${\hat{i}}_{k,\ell }(t)={c}_{i}({i}_{k,\ell }({t}_{0,\ell }:{t}_{f,\ell }))/{i}_{k,\ell }({t}_{0,\ell })$$

with *c*_*i*_ = − 1.675 A being a scaling constant to normalise against the training data (i.e., for the currents shown in Fig. 3), *t*_0,*ℓ*_ = 500 *s* being the starting time of the scaling current of Case *ℓ* (to reduce the effects of the harmonics at the beginning of the data), and *t*_*f*,*ℓ*_ being the final time.

## Supplementary information

Supplementary material

## Data Availability

The simulation and experimental datasets are available from the corresponding authors upon request.

## Code availability

The DFN-type simulation and post-processing codes that have been used to produce the results of this study are available by the corresponding authors upon request.
