# Supplementary material for: Detecting faulty lithium-ion cells in large-scale parallel battery packs using current distributions
Source: Commun Eng. 2026 Jan 21;5:17. doi: 10.1038/s44172-025-00543-x (PMC12823614; doi:10.1038/s44172-025-00543-x)
Supplement: Supplementary file 1 — Supplementary material [file 44172_2025_543_MOESM1_ESM.pdf]

Supplementary Information: Detecting faulty lithium-ion cells in large-scale parallel battery packs using current distributions

Pierre Lambert[1,2], Ross Drummond*[3], Eloise C. Tredenick[1] and Stephen R. Duncan[1]

[1]Department of Engineering Science, University of Oxford, OX1 3PJ, Oxford, UK
[2]École centrale de Nantes,1 Rue de la Noë, Nantes, 44300, France
[3]Department of Automatic Control and Systems Engineering, University of Sheffield, Sheffield, S1 3JD, UK

SI1: Parallel connected DFN models

Here the equations of the Doyle-Fuller-Newman (DFN) electrochemical model for a lithium-ion battery? are expressed in a form that allows the computation of the parallel branch currents to be applied. This formulation allows parallel connected packs of DFN models to be simulated. The main feature of this analysis is the treatment of the DFN model's voltage equation as the sum of linear series resistance term and a nonlinear term which is a function of the model's dynamical states (here, a state refers to a spatio-temporal variable with a time derivative in the model equations).

For this purpose, and with reference to Tables S1, S2 and S3 for the variable and parameter definitions and SI2 for the model boundary conditions, the DFN model equations for an electrode are

$$\frac{\partial u_s(x,r,t)}{\partial t} = \frac{\partial}{\partial r}\left(D_s \frac{\partial u_s(x,r,t)}{\partial r}\right), \tag{S1a}$$

$$\varepsilon \frac{\partial c_e(x,t)}{\partial t} = \frac{\partial}{\partial x}\left(D_e \frac{\partial c_e(x,t)}{\partial x}\right) + \frac{a_s(1-t^+)}{\mathscr{F}} j(x,t), \tag{S1b}$$

$$\frac{i_e(x,t)}{\kappa_e} = \frac{\partial \phi_e(x,t)}{\partial x} - \omega \frac{\partial \ln(c_e(x,t))}{\partial x}, \tag{S1c}$$

$$\frac{\partial i_e(x,t)}{\partial x} = a_s j(x,t) + a_s C \frac{\partial(\phi_s(x,t) - \phi_e(x,t))}{\partial t}, \tag{S1d}$$

$$\frac{i_s(x,t)}{\sigma_s} = \frac{\partial \phi_s(x,t)}{\partial x}, \tag{S1e}$$

$$i(t) = i_s(x,t) + i_e(x,t), \tag{S1f}$$

$$c_s^{\text{surf}}(x,t) = R_s u_s(x,R_s,t) \tag{S1g}$$

$$\eta(x,t) = \phi_s(x,t) - \phi_e(x,t) - U(c_s^{\text{surf}}(x,t)), \tag{S1h}$$

$$i_0 = k\mathscr{F}(c_s^{\max} - c_s^{\text{surf}}(x,t))^{\alpha_a} c_s^{\text{surf}}(x,t)^{\alpha_c} c_e(x,t)^{\alpha_a}, \tag{S1i}$$

$$\phi_{\text{dl}}(x,t) = \phi_s(x,t) - \phi_e(x,t), \tag{S1j}$$

$$j(x,t) = 2\, i_0(x,t) \sinh\left(\frac{\mathscr{F}}{2RT}\eta(x,t)\right), \tag{S1k}$$

and, in the separator, the governing equations are

$$\varepsilon \frac{\partial c_e(x,t)}{\partial t} = \frac{\partial}{\partial x}\left(D_e \frac{\partial c_e(x,t)}{\partial x}\right), \tag{S2a}$$

$$\frac{i(t)}{\kappa_e} = \frac{\partial \phi_e(x,t)}{\partial x} - \omega \frac{\partial \ln(c_e(x,t))}{\partial x}. \tag{S2b}$$

In the above, the k indices defining the cell number in the pack have been removed to simplify the notation, Equation S1a describes ion diffusion within the active particles, Equation S1b & Equation S2a are for ion diffusion in the liquid electrolyte, Equation S1h-Equation S1f define some of the variables from Table S3 and Equation S1d is the divergence equation which has been adapted to include double-layer dynamics using the approach of Ong and Newman.? Since the time constant of double-layer dynamics are fast, these dynamics are typically neglected from electrochemical battery models. However, they are required here as they give the necessary time derivative that allows the solid and liquid potentials $\phi_s(x,t)$ and $\phi_e(x,t)$ to be defined as dynamical states in the model. This is a crucial feature of the proposed analysis, and follows in the direction of Drummond and Duncan.? With these extra, fast dynamics, the equivalent $f(x_k(t))$ for the DFN model can be expressed in

*Email: ross.drummond@sheffield.ac.uk

terms of the model's states and so the methodology can then be directly applied to compute the parallel branch currents. Thus, by adding more physics to the DFN model through the addition of double-layer dynamics, the following analysis demonstrates how parallel connections of DFN models can be efficiently modelled.

Simulating a parallel pack of DFN models requires resolving Kirchhoff's equations and doing that requires understanding the model's voltage equation. For the DFN model, the voltage is defined as the potential difference in the solid phase of the electrode between each current collector, as in

$$V(t) = \phi_s(L,t) - \phi_s(0,t) + R_{\mathrm{ctc}} i(t). \tag{S3}$$

Using Equation S1j and Equation S1c, this expression can be re-written as

$$\begin{aligned} V(t) &= \phi_s(L,t) - \phi_s(0,t) + R_{\mathrm{ctc}} i(t) && \text{(S4a)}\\ &= \phi_{\mathrm{dl}}(L,t) - \phi_{\mathrm{dl}}(0,t) + \phi_{\mathrm{e}}(L,t) - \phi_{\mathrm{e}}(0,t) + R_{\mathrm{ctc}} i(t) && \text{(S4b)}\\ &= \phi_{\mathrm{dl}}(L,t) - \phi_{\mathrm{dl}}(0,t) + \int_0^L \frac{\partial \phi_{\mathrm{e}}(x,t)}{\partial x}\, dx + R_{\mathrm{ctc}} i(t), && \text{(S4c)}\\ &= \phi_{\mathrm{dl}}(L,t) - \phi_{\mathrm{dl}}(0,t) + \int_0^L \frac{i_e(x,t)}{\kappa_{\mathrm{e}}} + \omega \frac{\partial \ln(c_e(x,t))}{\partial x}\, dx + R_{\mathrm{ctc}} i(t), && \text{(S4d)} \end{aligned}$$

where the integral is evaluated across the whole domain (as in over the anode defined as $\Omega_n = [0, L_n]$, separator defined as $\Omega_s = [L_n, L_n + L_s]$ and cathode $\Omega_p = [L_n + L_s, L]$).

By combining Equations (S1j) (S1e) and (S1f), the ionic current can be written as

$$\begin{aligned} \frac{i_{\mathrm{e}}(x,t)}{\kappa_{\mathrm{e}}} &= \frac{\partial \phi_{\mathrm{e}}(x,t)}{\partial x} - \omega \frac{\partial \ln(c_{\mathrm{e}}(x,t))}{\partial x}, && \text{(S5)}\\ &= -\frac{\partial \phi_{\mathrm{dl}}(x,t)}{\partial x} + \frac{\partial \phi_{\mathrm{s}}(x,t)}{\partial x} - \omega \frac{\partial \ln(c_{\mathrm{e}}(x,t))}{\partial x}, && \text{(S6)}\\ &= -\frac{\partial \phi_{\mathrm{dl}}(x,t)}{\partial x} + \frac{i(t) - i_e(x,t)}{\sigma_{\mathrm{s}}} - \omega \frac{\partial \ln(c_{\mathrm{e}}(x,t))}{\partial x}, && \text{(S7)} \end{aligned}$$

or

$$\left(\frac{1}{\kappa_{\mathrm{e}}} + \frac{1}{\sigma_{\mathrm{s}}}\right) i_e(x,t) = -\frac{\partial \phi_{\mathrm{dl}}(x,t)}{\partial x} + \frac{i(t)}{\sigma_{\mathrm{s}}} - \omega \frac{\partial \ln(c_{\mathrm{e}}(x,t))}{\partial x}. \tag{S8}$$

In the electrode domains Ω_p, and Ω_n, the electrolyte current can then be expressed as

$$i_{\mathrm{e}}(x,t) = -\left(\frac{\sigma_{\mathrm{s}} \kappa_{\mathrm{e}}}{\sigma_{\mathrm{s}} + \kappa_{\mathrm{e}}}\right) \frac{\partial \phi_{\mathrm{dl}}}{\partial x} - \left(\frac{\omega \sigma_{\mathrm{s}} \kappa_{\mathrm{e}}}{\sigma_{\mathrm{s}} + \kappa_{\mathrm{e}}}\right) \frac{\partial \ln(c_{\mathrm{e}}(x,t))}{\partial x} + \frac{\kappa_{\mathrm{e}} i(t)}{\sigma_{\mathrm{s}} + \kappa_{\mathrm{e}}},\ x \in \Omega_k, k \in \{p, n\}, \tag{S9a}$$

whereas in the separator domain Ω_s, since all the current is carried by the electroylte's ions, it is simply

$$i_{\mathrm{e}}(x,t) = i(t),\ x \in \Omega_s. \tag{S9b}$$

Finally, substituting this expression back into the voltage term of Equation S4d gives

$$\begin{aligned} V(t) &= \phi_{\mathrm{dl}}(L,t) - \phi_{\mathrm{dl}}(0,t) \\ &+ \int_{\Omega_n} \frac{\omega \kappa_{\mathrm{e}}}{\sigma_{\mathrm{s}} + \kappa_{\mathrm{e}}} \frac{\partial \ln(c_{\mathrm{e}}(x,t))}{\partial x} - \frac{\sigma_{\mathrm{s}}}{\sigma_{\mathrm{s}} + \kappa_{\mathrm{e}}} \frac{\partial \phi_{\mathrm{dl}}}{\partial x} + \frac{i(t)}{\sigma_{\mathrm{s}} + \kappa_{\mathrm{l}}}\, dx \\ &+ \int_{\Omega_p} \frac{\omega \kappa_{\mathrm{e}}}{\sigma_{\mathrm{s}} + \kappa_{\mathrm{e}}} \frac{\partial \ln(c_{\mathrm{e}}(x,t))}{\partial x} - \frac{\sigma_{3}}{\sigma_{\mathrm{s}} + \kappa_{\mathrm{e}}} \frac{\partial \phi_{\mathrm{dl}}}{\partial x} + \frac{i(t)}{\sigma_{\mathrm{s}} + \kappa_{\mathrm{e}}}\, dx \\ &+ \int_{\Omega_s} \omega \frac{\partial \ln(c_{\mathrm{e}}(x,t))}{\partial x} + \frac{i(t)}{\kappa_{\mathrm{e}}}\, dx + R_{\mathrm{ctc}} i(t). \end{aligned} \tag{S10}$$

With this equation, the voltage of the DFN model has now been expressed in the form considered in the text, with a series resistance

$$r = R_{\mathrm{ctc}} + \int_{\Omega_n} \frac{1}{\sigma_{\mathrm{s}} + \kappa_{\mathrm{e}}}\, dx + \int_{\Omega_p} \frac{1}{\sigma_{\mathrm{s}} + \kappa_{\mathrm{e}}}\, dx + \int_{\Omega_s} \frac{1}{\kappa_{\mathrm{e}}}\, dx,$$

being the integrated inverse of the sum of the ionic and electronic conductivities in both the anode, cathode and separator. The remaining terms of Equation S10 are then collected together, to give

$$\begin{aligned} f = &\phi_{\mathrm{dl}}(L,t) - \phi_{\mathrm{dl}}(0,t) + \int_{\Omega_n} \frac{\omega \kappa_{\mathrm{e}}}{\sigma_{\mathrm{s}} + \kappa_{\mathrm{e}}} \frac{\partial \ln(c_{\mathrm{e}}(x,t))}{\partial x} - \frac{\sigma_{\mathrm{s}}}{\sigma_{\mathrm{s}} + \kappa_{\mathrm{e}}} \frac{\partial \phi_{\mathrm{dl}}}{\partial x}\, dx \\ &+ \int_{\Omega_p} \frac{\omega \kappa_{\mathrm{e}}}{\sigma_{\mathrm{s}} + \kappa_{\mathrm{e}}} \frac{\partial \ln(c_{\mathrm{e}}(x,t))}{\partial x} - \frac{\sigma_{\mathrm{s}}}{\sigma_{\mathrm{s}} + \kappa_{\mathrm{e}}} \frac{\partial \phi_{\mathrm{dl}}}{\partial x}\, dx + \int_{\Omega_s} \omega \frac{\partial \ln(c_{\mathrm{e}}(x,t))}{\partial x}\, dx. \end{aligned}$$

Crucially, this expression is now a function of the model's states, since the double-layer dynamics in Equation S1d give a time derivative for the double-layer potential $\phi_{\mathrm{dl}}(x,t)$.

With the voltage now in a similar form that considered in the text, in the sense that the voltage is a series resistance term plus a nonlinear function of the model states, the same framework can be directly applied to compute the branch currents.

SI2: Parameters and boundary conditions

The considered DFN model boundary conditions are as follows. Define the points at the current collector/electrode interfaces as $\Gamma_{\text{cc}\,|\,\text{el}}$. At this interface, then

$$i_{\mathrm{s}}(\Gamma_{\text{cc}\,|\,\text{el}},t) = i(t), \tag{S11a}$$

$$i_{\mathrm{e}}(\Gamma_{\text{cc}\,|\,\text{el}},t) = 0, \tag{S11b}$$

$$\left.\frac{\partial c_{\mathrm{e}}(x,t)}{\partial x}\right|_{x=\Gamma_{\text{cc}\,|\,\text{el}}} = 0. \tag{S11c}$$

Similarly, define the poitns at the separator/electrode interface as $\Gamma_{\text{sep}\,|\,\text{el}}$. At this interface, the boundary conditions are

$$i_{\mathrm{s}}(\Gamma_{\text{sep}\,|\,\text{el}},t) = 0, \tag{S12a}$$

$$i_{\mathrm{e}}(\Gamma_{\text{sep}\,|\,\text{el}},t) = i(t), \tag{S12b}$$

$$D_{\mathrm{e}}^{\mathrm{L}}\frac{\partial c_{\mathrm{e}}(x,t)}{\partial t}\bigg|^{\mathrm{L}}_{x=\Gamma_{\text{sep}\,|\,\text{el}}} = D_{\mathrm{e}}^{\mathrm{R}}\frac{\partial c_{\mathrm{e}}(x,t)}{\partial t}\bigg|^{\mathrm{R}}_{x=\Gamma_{\text{sep}\,|\,\text{el}}}. \tag{S12c}$$

In Equation S12c, the superscripts L and R denote the derivative pointing to the left and right at this boundary while $D_{\mathrm{e}}^{\mathrm{L}}$ and $D_{\mathrm{e}}^{\mathrm{R}}$ denote the values of the electrolyte diffusivity in the left and right domains of this interface, respectively. The boundary conditions for the active particles are

$$u_{\mathrm{s}}(x,0,t) = 0, \tag{S13a}$$

$$\frac{1}{R_{\mathrm{s}}}\left.\frac{u_{\mathrm{s}}(x,r,t)}{\partial r}\right|_{r=R_{\mathrm{s}}} = \frac{u_{\mathrm{s}}(x,R_{\mathrm{s}},t)}{{R_{\mathrm{s}}}^2} - \frac{j(x,t)}{\mathscr{F}D_{\mathrm{s}}}. \tag{S13b}$$

The diffusion coefficients and electrolyte conductivies were parameterised against data[?] and set to be functions of the electrolyte concentration, c_{e}, (R^2 values of 99.99% and 99.97%) according to

$$D_{\mathrm{s,k}} = (8.4\times10^{-9}\exp(-11.3\;c_{\mathrm{s,n}}/c_{\mathrm{s,n,max}}) + 8.2\times10^{11})\times10^{-4}, \quad \mathrm{k} = n \tag{S14a}$$

$$D_{\mathrm{e,k}} = D_{\mathrm{e,bulk}}\;\varepsilon_{\mathrm{e,k}}{}^{b_{\mathrm{k}}}, \;\; \mathrm{k}\in\{\mathrm{n,s,p}\}, \tag{S14b}$$

$$\kappa_{\mathrm{e,k}} = \kappa_{\mathrm{e,bulk}}\;\varepsilon_{\mathrm{e,k}}{}^{b_{\mathrm{k}}}, \;\; \mathrm{k}\in\{\mathrm{n,s,p}\}, \tag{S14c}$$

$$D_{\mathrm{e,bulk}}(c_{\mathrm{e}}) = -7.55\times10^{-21}\;c_{\mathrm{e}}^3 + 8.231\times10^{-17}\;c_{\mathrm{e}}^2 - 3.401\times10^{-13}\;c_{\mathrm{e}} + 5.464\times10^{-10}, \tag{S14d}$$

$$\kappa_{\mathrm{e,bulk}}(c_{\mathrm{e}}) = 5.746\times10^{-18}\;c_{\mathrm{e}}^5 - 1.025\times10^{-13}\;c_{\mathrm{e}}^4 + 7.082\times10^{-10}\;c_{\mathrm{e}}^3 - 2.236\times10^{-6}\;c_{\mathrm{e}}^2 + 0.00273\;c_{\mathrm{e}} - 0.003, \tag{S14e}$$

$$a_s = \frac{3\;(1-\varepsilon_{\mathrm{e}}-\varepsilon_{\mathrm{CBD}})}{R_{\mathrm{s}}}, \tag{S14f}$$

$$\omega = \frac{2\;(1-t^{+})\;R\,T}{F}. \tag{S14g}$$

with the indice k indicating whether the parameter is set for the anode (k = n), separator (k= s) or cathode (k = p). Finally, a reference potential of $\phi_{\mathrm{e}}(0,t) = 0$ was set.

Parameter	Description	Unit	NMC	Sep	LiC6	Source
b	Bruggeman tortuosity factor	-	2	1.5	1.64	-
$c_{\mathrm{s},0}$	Initial concentration of Li ions in solid particles	mol/m^3	13,149	-	31,282	-
$c_{\mathrm{s,max}}$	Maximum concentration of Li ions in solid particles	mol/m^3	48,700	-	31,920	[?]
D_{s}	Diffusivity of Li ions in solid	m^2/s	3.995×10^{-14}	-	Eq. (S14a)	[?]
k	Reaction rate constant	$\mathrm{m^{2.5}s^{-1}mol^{-0.5}}$	9×10^{-12}	-	2.33×10^{-10}	- ,[?]
L_{k}	Thickness	μm	65	16	76	Exp
R_{s}	Radius of particle	μm	5	-	13.7	,[?] [?]
$\varepsilon_{\mathrm{CBD}}$	Carbon binder domain volume fraction	-	0.12	-	0.118	-
ε_{e}	Electrolyte volume fraction	-	0.3	0.45	0.3	-
σ_{s}	Electronic conductivity in solid	S/m	5	-	14	Exp,[?]

Table S1: Model parameters for NMC622 and graphite LiC6 anode with the subscript k ∈ {p,s,n} defining the cathode, separator and anode regions, respectively. The 'Exp' values were measured from experiments while the other parameter values were found elsewhere.[?]

Parameter	Description	Unit		Source
A	Electrode cross-sectional area	m^2	1.54×10^{-4}	
c_{e0}	Initial concentration of Li ions in electrolyte	mol/m^3	1,000	Exp
C_{cell}	Cell capacity (cathode)	mAh/g	175	Exp
$\mathscr{F}$	Faraday constant	sA/mol	96485.332	[?]
I	Cell current density	A/m^2	$I = I_c/A$	
I_c	Applied cell current at 1C	mA	5.27	Exp
R	Universal gas constant	J/K/mol	8.314463	[?]
R_{ctc}	Contact resistance	$\Omega\ m^2$	5.7×10^{-4}	
T	Constant absolute reference temperature	K	293.15 (20°C)	Exp
t^+	Transference number of the electrolyte	-	0.37	[?]
C	Double layer capacitance	F/m^2	0.2	[?]

Table S2: Cell parameters for the DFN model.

Parameter	Description	Unit
a_s	Reaction surface area per volume	1/m
c_e	Concentration of lithium in the liquid electrolyte	mol/m^3
c_s	Concentration of lithium in the solid particles	mol/m^2
u_s	Scaled Li concentration in the solid particles	mol/m^3
D_e	Diffusivity function in the electrolyte	m^2/s
$D_{e,bulk}$	Bulk diffusivity function in the electrolyte	m^2/s
j	Butler-Volmer reaction rate	A/m^2
i_0	Exchange reactions	A/m^2
r	Particle radius	m
t	Time	s
U	Open circuit potential (OCP)	V
v	Voltage	V
x	Direction through cell	m
Ω_k	Anode/cathode/separator spatial domain	m^2
κ_e	Ionic conductivity function in the electrolyte	S/m
$\kappa_{e,bulk}$	Bulk ionic conductivity function in the electrolyte	S/m
η	Local overpotential	V
ϕ_s	Potential in the solid particles	V
ϕ_e	Potential in the liquid electrolyte	V
ϕ_{dl}	Potential difference between solid particles and liquid electrolyte	V

Table S3: Variables of the DFN model with the subscript $k \in \{p,s,n\}$ corresponding to the cathode, separator and anode compartments.
